# Enhanced Removal of Antibiotic Sulfachloropyridazine in Water Using Sodium Percarbonate Activated by Ozone: Mechanism, Degradation Pathway, and Toxicity Assessment

**DOI:** 10.3390/toxics14010073

**Published:** 2026-01-13

**Authors:** Junqi Jia, Wenhao Wang, Yulong Liang, Zhangbin Pan, Congcong Li

**Affiliations:** 1 CAUPD (Beijing) Planning & Design Consultants Co., Ltd., Beijing 100044, China; jiajunqi1997@126.com; 2 Jinan Municipal Engineering Design & Research Institute Co., Ltd., Jinan 266100, China; 18366185082@163.com; 3 College of Municipal and Environmental Engineering, Shandong Jianzhu University, Jinan 250101, China; 13370511140@163.com; 4 The People’s Government of Dongzhuang Town, Tai’an 271400, China; 5 Shandong Province City Water Supply and Drainage Water Quality Monitoring Center, Jinan 250021, China; 6 School of Water Conservancy and Environment, University of Jinan, Jinan 250022, China

**Keywords:** sulfachloropyridazine (SCP), sodium percarbonate (SPC), ozone (O_3_), advanced oxidation process (AOP)

## Abstract

Antibiotics have become an integral part of human life and production. The presence of sulfachloropyridazine (SCP), one of the most ubiquitous antibiotics, in water has been a growing concern owing to its long persistence and the difficulty in removing it by conventional water treatment processes. This study introduced ozone (O_3_)-activated sodium percarbonate (SPC) as an innovative technique of advanced oxidation processes (AOPs), and the degradation of SCP from water by this method was thoroughly investigated. The impact of a variety of parameters, such as the dosage of SPC, the dosage of O_3_, the pH value, and water matrix constituents, on the removal of SCP was evaluated with regard to the pseudo-first-order kinetic model. It was found that the removal effectiveness of SCP improved initially and then decreased with the rising dosage of SPC, with an optimal SPC dose achieved at 20 mg/L. Moreover, •OH, $O_{2}^{•-}$ and ^1^O_2_ played important roles during SCP degradation based on radical quenching tests and electron paramagnetic resonance (EPR) tests. The SCP degradation pathways were predicted using density functional theory (DFT), which primarily involves the cleavage of S-C or S-N bonds and Smiles-type rearrangements, accompanied by hydroxylation. Furthermore, the toxicity of degradation intermediates was evaluated by the ECOSAR 1.1 software in terms of acute toxicity and chronic toxicity, and most of them exhibited lower levels of toxicity. The results can expand the research scope of SPC and reveal significant insights for SPC’s application in controlling antibiotic contamination.

## 1. Introduction

Antibiotics are widely used in veterinary and human medicine due to their low cost and effectiveness in disease prevention, whereas many are inevitably released into the various natural environments, such as water, soil, and sediment, causing hazards to ecosystems and humans. Sulfachloropyridazine (SCP) is one of the most commonly prescribed antibiotics because of its broad-spectrum properties against chlamydia, Gram-positive bacteria, and Gram-negative aerobic bacteria. Some researchers have previously tested conventional techniques, such as biodegradation, adsorption, and electrochemical processes, for removing antibiotics from water and wastewater, revealing that the application of these techniques faces challenges, as they are generally time-consuming, comparatively expensive, and struggle to handle the adsorbent carrier [1,2,3,4]. Therefore, it is imperative to develop both effective and efficient methods for the removal of antibiotics.

Compared to conventional treatment processes, advanced oxidation processes (AOPs) demonstrate greater potential for degrading and mineralizing recalcitrant and toxic organic pollutants in water [5]. Ozone oxidation decomposes to produce hydroxyl radicals (•OH) in water, which are characterized as non-selective oxidizing substances with extraordinary oxidizing power. Nevertheless, it has some drawbacks, including the low O_3_ utilization rate, high dosage, selective oxidation, and low free radical generation efficiency [6,7]. Thus, it is necessary to modify the ozonation process and improve the oxidation capacity, thereby enhancing the removal of organic pollutants. In recent years, a series of studies on the optimization of ozonation processes has emerged based on single ozonation, often involving O_3_/UV [8], O_3_/catalyst [9], sonication–ozonation [10], O_3_/hydroxylamine [11], and O_3_/H_2_O_2_ [12]. Guo [13] and Malik [14] applied homogeneous catalyzed ozonation with transition metals (Ti^2+^, Co^2+^, Ni^2+^, Zn^2+^, Cu^2+^, Mn^2+^, Fe^2+^, and Fe^3+^) to generate •OH, achieving a high degree of organic pollutant degradation. The large-scale use of homogeneous catalytic ozonation is as yet limited by the possible secondary pollution caused by transition metal ions in solution, despite its strong catalytic potency. As for combined ozonation technologies, the O_3_/H_2_O_2_ approach has been recognized as one of the most efficient ways to treat refractory organic matter, as H_2_O_2_, a comparatively “green” reagent, can promote the decomposition of O_3_ to greatly yield •OH [15]. But considering that H_2_O_2_ is an explosive, hazardous substance that is difficult to transport and store, there are significant risks and restrictions for its practical applications.

Attention has been paid to sodium percarbonate (SPC, Na_2_CO_3_·1.5H_2_O_2_), an addition compound of sodium carbonate and hydrogen peroxide, as an environmentally benign reagent with a high oxidation potential (1.8 eV) [16,17,18]. Sodium percarbonate contains an active oxygen content equivalent to that of 27.5% H_2_O_2_. In comparison to H_2_O_2_, SPC is eco-friendly, non-toxic, non-corrosive, and easily transportable or storable [19,20]. As a green oxidant with excellent water solubility, it has been extensively utilized as a bactericide or bleaching agent in the papermaking, laundering, and health industries. Numerous studies have demonstrated that SPC may exhibit a strong ability, under certain activation, to decompose organic contaminants, thereby replacing H_2_O_2_ [21,22]. Guo et al. [23] reported that SPC, activated by O_3_, showed enhanced degradation of sulfamethoxazole in water, as the removal rate rose by 16.4%. Yu et al. revealed that a combined system of O_3_/SPC resulted in a more efficient oxidation process than O_3_ alone due to the substantial generation of reactive species (such as •OH and ${\mathrm{CO}}_{3}^{•-}$) [24]. To our knowledge, limited studies have been conducted about the removal of SCP utilizing the combined process of O_3_/SPC. Consequently, this study innovatively combines O_3_ and SPC to degrade SCP, fills the research gap in SCP degradation by the O_3_/SPC system, and overcomes the practical limitations of conventional O_3_-enhanced oxidation technologies.

Herein, degradation of antibiotic SCP in drinking water using SPC activated by O_3_ was investigated. The effects of O_3_ and SPC doses, water matrix components, and pH value on SCP removal were evaluated. Moreover, both free radical quenching experiments and electron paramagnetic resonance (EPR) tests were performed to discover reactive oxygen species (ROS) and examine their synergistic effects. The intermediates generated during the reaction were detected and analyzed by gas chromatography mass spectrometry (GC-MS), and the possible degradation pathway of SCP was speculated. The results provide new insights for future applications of the O_3_/SPC system in controlling the risk of antibiotics in drinking water, as a supplement for the conventional water treatment processes.

## 2. Materials and Methods

### 2.1. Chemicals and Reagents

Sodium dihydrogen phosphate dihydrate, sodium thiosulfate, *p*-benzoquinone, 1,4-diazabicyclo [2.2.2] octane, disodium hydrogen phosphate (dodecahydrate), dimethyl sulfoxide, and hydrogen peroxide aqueous solution (30%) were bought from Aladdin’s Reagent Co., Ltd. (Shanghai, China). Sodium indigo disulfate and the spin trap reagents DMPO (C_6_H_11_NO, ≥97%) and TEMP (C_9_H_19_N, ≥97%) were bought from Aladdin’s Reagent Co., Ltd. Sodium and sodium indigo disulfate, sulfachloropyridazine (analytically pure), and sodium sulfate were purchased from McLean. Methanol (chromatographically pure) was purchased from Merck & Co., Ltd. (Darmstadt, Germany). Sodium hypochlorite and sodium nitrite (analytically pure) were purchased from Tianjin Komeo Chemical Reagent Co., Ltd. (Tianjin, China). Sodium percarbonate, with an active oxygen content of 12–14%, was purchased from McLean Co., Ltd. (Shanghai, China). Sodium chloride (analytically pure) was obtained from Sinopharm Chemical Reagent Co., Ltd. (Shanghai, China). Potassium iodide (analytically pure) was bought from Chron Chemicals Co., Ltd. (Chengdu, China). Ultrapure water, produced by a Millipore System (Billerica, MA, USA), was used to prepare all the aqueous solutions.

### 2.2. Experimental Methods

The experimental setup mainly consisted of an ozone generator, a glass vessel as the reactor, and a conical flask for collecting the exhaust gas. The diagram of the experimental configuration is presented in Figure S1. The O_3_ was generated by a Changqing CQ-802S ozone generator (Jinan, China). A rotating flowmeter was set in the middle of the delivery tube connecting the ozone generator and the reactor to regulate the O_3_ injection. An aeration device was used to bubble O_3_, with aeration duration set to 10 min, and different ozone dosages were controlled by adjusting the generator settings and gas flow rates. Afterwards, 1 g/L SPC stock and 100 mg/L SCP stock were prepared in advance and added to the reactor in the necessary amounts. The pH value of the solution was adjusted using the phosphate buffer. To ensure the solution was well mixed throughout the reaction, a magnetic stirrer was placed within the reactor. Samples were taken at 0, 2, 4, 6, 8, and 10 min during the reaction using sufficient sodium thiosulfate to quench oxidants, and the concentration of SCP residual was then measured. The exhaust gas was then collected by the tail gas flask and quenched by the potassium iodide solution.

### 2.3. Analytical Methods

#### 2.3.1. Basic Water Quality Analysis

The pH value of the solution within the reactor was measured using a Mettler Toledo pH analyzer (Zurich, Switzerland). The concentration of ozone in the solution was quantified by using a Tu-1810 Ultraviolet-visible (UV−vis) spectrophotometer (Beijing, China) by a modified indigo method.

#### 2.3.2. Chromatographic Analysis

The concentration of SCP was determined using Waters ultra-high-performance liquid chromatography coupled with a triple quadrupole mass spectrometer (Milford, MA, USA). The liquid chromatography was programmed to run for eight minutes with the following mobile phases: A, 0.1% formic acid aqueous solution; B, methanol; C, mixture of methanol and acetonitrile (50%:50%) with 0.1% formic acid; and D, ultrapure water. The injection volume of the sample was fixed at 10 μL. The chromatographic separation was conducted with a Waters ACQUITY UPLC BEH C18 analytical column (100 × 2.1 mm, 3 μm) at a constant flow rate of 0.3 mL/min. The initial gradient was 0–4 min, with 75% A and 25% C; from 4 to 5.1 min, A was decreased from 75% to 40% and B was increased from 25% to 60%, and from 5.1 to 6 min, A and B were switched back to 75% and 25%, respectively. The mass spectrometry was set as an electrospray ionization source in positive ion mode, with a filling volume of 250 μL. The rinse program was achieved with the mixture of 90% methanol and 10% ultrapure water.

#### 2.3.3. EPR Measurements

Electron paramagnetic resonance (EPR) measurements were performed, combined with DMPO and TEMP which were utilized as radical spin traps. EPR spectra were measured using a Bruker A300 spectrometer (Billerica, MA, USA), equipped with a liquid helium low-temperature cryostat. Sample solutions (100 mM) were tested under microwave power ranging from 1 to 20 mW, with a field modulation of 100 kHz and a modulation amplitude of 1 G. Spectral simulations implemented in EasySpin (v5.2.23) were employed to extract the g-values.

### 2.4. Computational Methods

#### 2.4.1. Reaction Kinetics Modeling

A pseudo-first-order kinetic model was established to analyze *SCP* degradation kinetics, as shown below (Equations (1) and (2)):

(1) $$-\frac{d\left[SCP\right]}{dt}=k[SCP]$$

(2) $$In\frac{Co}{Ct}=kt$$

where [*SCP*] is the concentration of sulfachloropyridazine (mg/L); *t* is the reaction time (min); *k* is the observed pseudo-first-order rate constant (min^−1^); *Co* is the initial concentration of *SCP* (mg/L); and *Ct* is the remaining concentration of *SCP* at time *t* (mg/L).

#### 2.4.2. DFT Analysis and Toxicity Assays

The molecular structure of SCP was analyzed based on the density functional theory (DFT) by using the B3LYP Density Functional Method with the 6-31G(d) basis, which was performed on Material Studio (MS, version 4.2.1) with CASTEP and Dmol 3 module.

The toxicity levels of degradation intermediates, including acute toxicity and chronic toxicity, were assessed by the ECOSAR software focused on aquatic organisms: Daphnia magna, fish, and green algae.

## 3. Results and Discussion

### 3.1. Effect of Reaction Parameters on Degradation of SCP by O_3_/SPC

#### 3.1.1. Effect of O_3_ Dosage on SCP Removal

**Figure 1:**
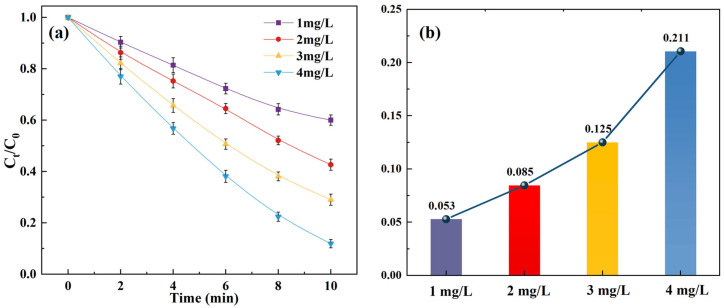
Effect of O_3_ dosage on the SCP removal: (**a**) degradation curves; (**b**) kinetic constants. Experimental conditions: [SCP]_0_ = 1 mg/L, [SPC] = 20 mg/L, and pH = 7 (10 mM phosphate buffer).

To investigate the effect of ozone dosage on the SCP removal, Figure 1 and Figure S2 display the SCP removal of the O_3_/SPC system under varying O_3_ doses (1, 2, 3, and 4 mg/L).

It can be perceived that with an increase in O_3_ dosage, both the removal efficiency and effectiveness displayed upward trends, as 40.0%, 57.3%, 71.0%, and 88.1% of SCP were degraded within the O_3_/SPC system with O_3_ doses of 1, 2, 3, and 4 mg/L after 10 min reactions. Meanwhile, the increase in O_3_ dosage, from 1 mg/L to 4 mg/L, resulted in a higher kinetic constant, which increased from 0.053 to 0.211 min^−1^ correspondingly (Figure 1b). The ozone dosage, in the range of 1 to 4 mg/L, was generally practical, which has been applied in several relevant studies previously [25,26]. At the beginning of the reactions, the SPC was dissociated in water, generating hydrogen peroxide (H_2_O_2_) and sodium carbonate (Equation (3)) [27,28]. The interaction between the produced H_2_O_2_ and O_3_ led to the production of hydroxyl free radicals (•OH) (Equation (4)) [29]. Consequently, the increase in O_3_ dosing could have promoted the reaction between O_3_ and H_2_O_2_ to release •OH, which in turn generated other reactive species and also directly increased the effective contact reaction frequency between the formed •OH and the target antibiotic pollutant SCP [30], eventually enhancing SCP removal effectiveness and efficiency.

(3) $${Na}_{2}CO_{3}{\cdot 1.5H}_{2}O_{2}\to \;{Na}_{2}CO_{3}+{1.5H}_{2}O_{2}$$

(4) $${2O}_{3}+H_{2}O_{2}\to \;2•OH+{3O}_{2}$$

Moreover, the increase in the partial pressure of ozone in the gas phase could accelerate its transfer towards the liquid phase, which is utilized more effectively by the O_3_/SPC system than the O_3_ alone, as previously reported [24].

#### 3.1.2. Effect of SPC Dosage on SCP Removal

**Figure 2:**
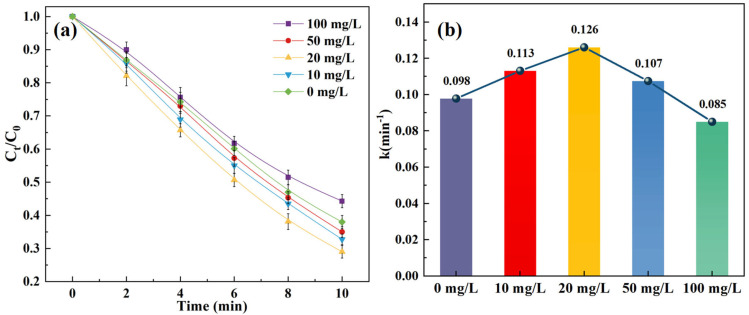
Effect of SPC dosage on the SCP removal: (**a**) degradation curves; (**b**) kinetic constants. Experimental conditions: [SCP]_0_ = 1 mg/L, [O_3_] = 3 mg/L, and pH = 7 (10 mM phosphate buffer).

Figure 2 and Figure S3 illustrate the effects of different SPC doses (0, 10, 20, 50, and 100 mg/L) on SCP removal in the O_3_/SPC system.

The results indicated that removal rates of SCP in the O_3_/SPC system were 62.6%, 67.3%, 71.8%, 66.5%, and 55.8% after 10 min at the initial SPC doses of 0, 10, 20, 50, and 100 mg/L, respectively. It is evident that the best removal rates and corresponding kinetic constants in this test were achieved at an SPC dose of 20 mg/L. The increase in SPC dosage at low levels, in the range of 0–20 mg/L, facilitated SPC degradation, which mainly resulted from the promoted generation of •OH with more H_2_O_2_ produced. Since the 10 mM phosphate buffer was applied, the alkaline condition was not further enhanced by the addition of SPC to further boost the •OH production. The impact of increasing SPC dosage was less significant on the overall degradation of target pollutant compared to a previous study [31], which used dilute sulfuric acid and sodium hydroxide to balance the pH level.

Moreover, it was observed that a further increase in SPC dosage, at the initial doses of 50 and 100 mg/L, hindered SCP removal, and a similar phenomenon had been noted previously [19,32,33]. This indicated that the excess SPC in water could overly produce substances that consume the •OH generated and abate the ability of the O_3_/SPC system to oxidize. It is surmised that the consumption of •OH is mainly involved with hydrogen peroxide, carbonate ions, and $HO_{2}^{-}$, a decomposition product of ozone in water, causing competition with the target pollutant SCP, which would become significant when SPC dosage reaches certain levels (Equations (5)–(9)). These reactions primarily result in products with lower oxidation potentials, which may be as reactive as with the pollutant SCP.

(5) $$H_{2}O_{2}+•OH\to \;HO_{2}^{•-}+H_{2}O$$

(6) $$HO_{2}^{-}+•OH\to \;HO_{2}^{•-}+{HO}^{-}$$

(7) $$CO_{3}^{2-}+•OH\to \;CO_{3}^{•-}+{HO}^{-}$$

(8) $$HCO_{3}^{-}+•OH\to \;CO_{3}^{•-}+H_{2}O$$

(9) $$H_{2}O_{2}+•OH\to \;O_{2}^{•-}+H_{2}O+H^{+}$$

#### 3.1.3. Effect of Solution pH on SCP Removal

**Figure 3:**
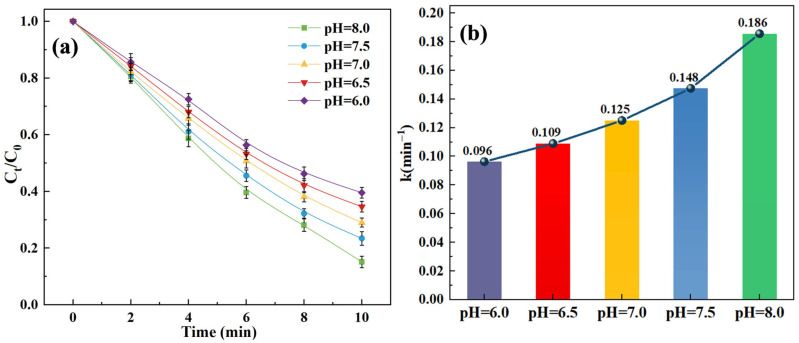
Effect of pH value on the SCP removal: (**a**) degradation curves; (**b**) kinetic constants. Experimental conditions: [SCP]_0_ = 1 mg/L, [O_3_] = 3 mg/L, and [SPC] = 20 mg/L.

The pH of the solution often plays a pivotal role in O_3_-based AOPs by affecting the decomposition of O_3_ and production of reactive oxygen species, such as •OH [34]. Thus, the influence of different pH levels (6.0, 6.5, 7.0, 7.5, 8.0) on the SPC degradation in the O_3_/SPC system was evaluated. As shown in Figure 3 and Figure S4, the alkaline condition was more favorable to the SCP removal in the O_3_/SPC system. As the solution pH was elevated by 10mM phosphate buffer from 6.0 to 8.0, the removal rate of SCP gradually increased from 60.5% to 84.9% after a 10 min reaction by O_3_/SPC, with the corresponding kinetic constant increasing from 0.096 to 0.186 min^−1^, respectively. This is consistent with previous studies on the degradation of organic contaminants by O_3_/H_2_O_2_ and O_3_/SPC systems, exhibiting optimal removal at alkaline conditions [27,35]. Analogous to O_3_/H_2_O_2_, the oxidation effectiveness and efficiency of the O_3_/SPC system were directly dependent upon the generation of •OH, and its production rate in the O_3_/H_2_O_2_ system is particularly slow under acidic conditions, where H_2_O_2_ mostly exists in molecular form, being less reactive with O_3_ compared to neutral or alkaline conditions [36]. Because H_2_O_2_ is more susceptible to ionization under neutral or alkaline conditions, it deprotonates to form ${\mathrm{HO}}_{2}^{•-}$, which is more reactive with ozone to generate •OH [37]. Therefore, when the pH increased from 6.0 to 8.0, the dominating oxidation pathway of SCP shifted from direct oxidation bsy O_3_ to indirect oxidation by •OH and its consequential reactive species. In addition, previous research indicated that the degradation of SCP was strongly dependent on pH, with the most significant decrease occurring in alkaline conditions under simulated sunlight [38].

### 3.2. Effect of Water Substrates on Degradation of SCP by O_3_/SPC

#### 3.2.1. Inorganic Anions in Water

Inorganic anions are commonly present in sources of drinking water and may influence the O_3_/SPC system [39]. In this section, the effects of common inorganic anions, including $NO_{2}^{-}$ and Cl^−^, were analyzed on SCP removal by the O_3_/SPC system.

**Figure 4:**
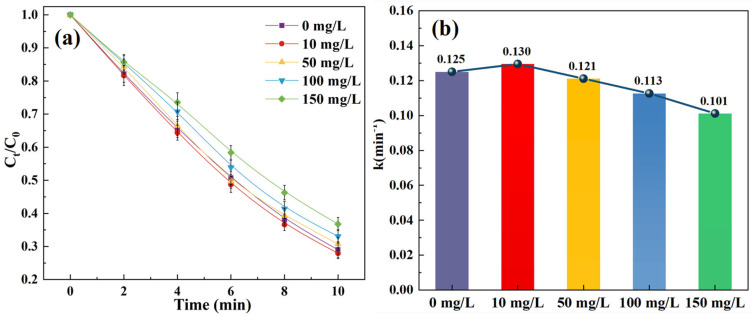
Effect of Cl^−^ on the removal of SCP: (**a**) degradation curves; (**b**) kinetic constants. Experimental conditions: [SCP]_0_ = 1 mg/L, [O_3_] = 3 mg/L, [SPC] = 20 mg/L, and initial pH = 7.

As a result, the SCP removal rates after 10 min reached 71.0%, 72.1%, 69.2%, 66.9%, and 63.2% for chloride concentrations of 0, 10, 50, 100, and 150 mg/L, respectively, and the variation in the kinetic constant followed the same pattern (Figure 4 and Figure S5). Notably, the SCP removal was suppressed at chloride concentrations exceeding 50 mg/L. As previously studied and interpreted, and cited and displayed in Equations (10)–(14), the chloride ions react with •OH to generate Cl•, with which a rapid backward reaction takes place, and the chloride ions can further react with Cl• to produce ${\mathrm{Cl}}_{2}^{•-}$ [40,41,42,43]. Arguably, the Cl• could be an important contributor to the degradation of certain pollutants and the •OH might even be regenerated, but with a sufficient amount of chloride ions in water, ≥50 mg/L in this case, the generation of ${\mathrm{Cl}}_{2}^{•-}$ and products of its consequential reactions, which are less reactive with contaminants, should be dominant, explaining the inhibitory effect on the overall removal effectiveness and efficiency of SCP.

(10) $${Cl}^{-}+•OH\to \;•Cl{OH}^{-}$$

(11) $$•Cl{OH}^{-}\to \;{Cl}^{-}+•OH$$

(12) $$•Cl{OH}^{-}+H^{+}\to \;Cl•+H_{2}O$$

(13) $$•Cl{OH}^{-}\to \;Cl•+{OH}^{-}$$

(14) $$Cl•+{Cl}^{-}\to \;{Cl}_{2}^{•-}$$

**Figure 5:**
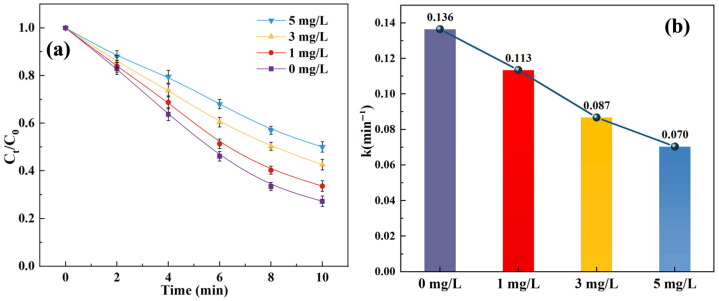
Effect of $NO_{2}^{-}$ on the removal of SCP: (**a**) degradation curves; (**b**) kinetic constants. Experimental conditions: [SCP]_0_ = 1 mg/L, [O_3_] = 3 mg/L, [SPC] = 20 mg/L, and initial pH = 7.

Figure 5 and Figure S6 reveal the effect of $NO_{2}^{-}$ on SCP removal by the O_3_/SPC system, indicating that $NO_{2}^{-}$ exhibited a significant inhibitory effect on the removal of SCP. For the $NO_{2}^{-}$ concentrations of 0, 1, 3, and 5 mg/L, 72.8%, 66.4%, 57.4%, and 50.0% of the SCP was removed after 10 min, respectively, with the kinetic constant declining correspondingly. As previously reported, $NO_{2}^{-}$ is considered a strong scavenger for •OH, with a higher rate constant $k_{•OH,NO_{2}^{-}}$ compared to other common inorganic ions [44]. Therefore, the reaction between $NO_{2}^{-}$ and •OH, even at a much smaller range of concentration of nitrite ions compared to those of chloride ions, would lead to a series of consequential reactions and comprise the degradation of SCP, as mainly displayed in Equations (15)–(18). Furthermore, $NO_{2}^{-}$ can be directly oxidized by O_3_ to generate $NO_{3}^{-}$ (Equation (19)).

(15) $${NO}_{2}^{-}+•OH\to \;{NO}_{2}•+{OH}^{-}$$

(16) $${NO}_{2}•+•OH\to \;ONOOH$$

(17) $${2NO}_{2}•\to \;N_{2}O_{4}$$

(18) $$N_{2}O_{4}+H_{2}O\to \;{NO}_{3}^{-}+2H^{+}+{NO}_{2}^{-}$$

(19) $${NO}_{2}^{-}+O_{3}\to \;{NO}_{3}^{-}+O_{2}$$

#### 3.2.2. Natural Organic Matter in Water

**Figure 6:**
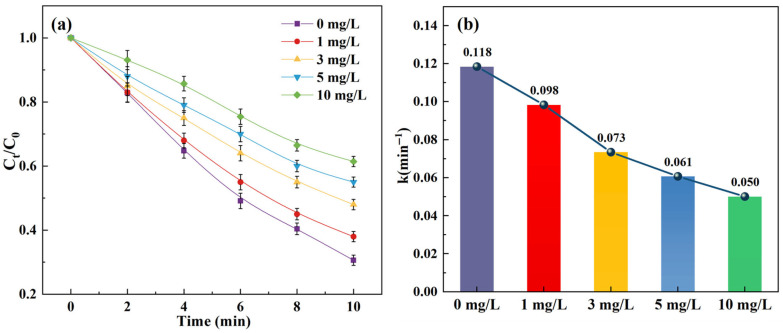
Effects of humic acid on the removal of SCP: (**a**) degradation curves; (**b**) kinetic constants. Experimental conditions: [SCP]_0_ = 1 mg/L, [O_3_] = 3 mg/L, [SPC] = 20 mg/L, and initial pH = 7.

In addition to inorganic ions, natural organic matter (NOM) is also ubiquitously present in water, influencing the removal of contaminants by oxidation systems [45,46]. Humic acid (HA), a common form of NOM, was used to evaluate the impact of NOM on the SCP removal. HA is a large molecule substance with complex structures, consisting of a variety of functional groups, such as phenols, carboxyl groups, and alkoxy groups [45]. Figure 6 and Figure S7 illustrate that the SCP removal effectiveness by the O_3_/SPC system decreases with increasing HA concentration, which is in agreement with previous research [24]. For HA concentrations of 0, 1, 3, 5, and 10 mg/L, SCP removal rates after 10 min were 69.4%, 62.0%, 52.0%, 45%, and 38.6%, respectively. Meanwhile, the kinetic constant declined from 0.018 to 0.050 min^−1^ as the concentration of HA increased from 0 to 5 mg/L. This salient inhibition was mainly ascribed to the competition for the oxidative reagents between HA and the target pollutant SCP, primarily inducing the oxidation cleavage process of HA [28,34].

### 3.3. Identification of Reactive Oxygen Species (ROS) Within O_3_/SPC

**Figure 7:**
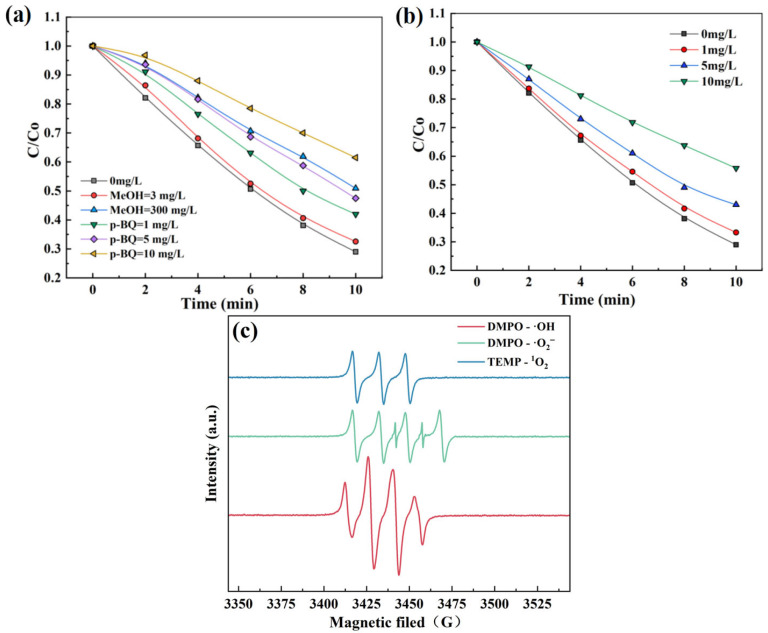
(**a**) Quenching effects of MeOH and BQ for radicals in the degradation of SCP by O_3_/SPC system; (**b**) quenching effects of DABCO for singlet oxygen in the degradation of SCP by O_3_/SPC system; (**c**) EPR spectra at a reaction time of 10 min in O_3_/SPC system.

As discussed in the previous sections, the oxidation efficiency and effectiveness of the O_3_/SPC system primarily depended on the generation of •OH, ehereas various reactive oxygen species (ROS) could have been generated subsequently, which might have contributed to the degradation of SCP as well. To identify the ROS and understand their role in the degradation of SCP by the O_3_/SPC system, free radical quenching experiments were conducted, with methanol (MeOH), *p*-benzoquinone (BQ), and 1,4-diazabicyclo [2.2.2] octane (DABCO) serving as scavenging agents. MeOH was chosen as the scavenger for •OH due to the high reactivity (9.7 × 10^8^ M^−1^ s^−1^), and BQ could react with superoxide radicals ($O_{2}^{•-}$) readily (0.9–1.0 × 10^9^ M^−1^ s^−1^), and could therefore be utilized as another scavenger [47]. In addition, singlet oxygen (^1^O_2_), which could be generated as an intermediate with a strong oxidation potential, was effectively trapped by DABCO [32,48]. As a result, the addition of three scavengers all led to the decline in the removal rate of SCP, indicating that •OH, $O_{2}^{•-}$, and ^1^O_2_ all played important roles in the degradation of SCP (Figure 7a,b). Furthermore, the removal rate of SCP decreased from 71.0% to 58.0%, 52.5%, and 38.5% within 10 min with the addition of BQ at 1, 5, and 10 mg/L, respectively, which implied that BQ exhibited the most significant quenching effect among three scavenging agents. ^1^O_2_ also served as an important contributor as the removal rate of SCP declined from 71.0% to 66.7%, 57.0%, and 44.1% in 10 min, with the dose of DABCO at 1, 5, and 10 mg/L, respectively. Whereas the quenching effect of MeOH was less substantial, since even dosing 300 mg/L MeOH, approximately tenfold the dose of BQ at 10 mg/L in molar ratio, resulted in a smaller decrease in the removal rate of SCP from 71.0% to 49.1%. Therefore, it is speculated that the majority of •OH generated in the O_3_/SPC system might be immediately consumed and lead to a series of sequential reactions, which could supersede the quenching by MeOH and produce multiple other ROS to react with SCP. Arguably, as recently debated, the quenching effect of a scavenger might not guarantee its contribution to pollutant degradation [41]. Nevertheless, the generation of these three reactive species within the O_3_/SPC system could still be implied.

To further validate the ROS identified by quenching experiments, EPR measurements combined with DMPO and TEMP, used as the radical trapping agents, were applied to confirm the type and relative intensity of radicals generated in the O_3_/SPC system. DMPO was used to trap superoxide radicals ($O_{2}^{•-}$) and hydroxyl radicals (•OH), and TEMP was used to trap singlet oxygen (^1^O_2_). A distinctive quadruple peak ratio of 1:2:2:1 was observed for DMPO-•OH, the DMPO-$O_{2}^{•-}$ showed a distinct 2:2:1:2:1:2 hexagonal line feature spectrum, and the TEMP-^1^O_2_ displayed a distinct 1:1:1 quadruple line feature spectrum (Figure 7c). Based on these results, it is verified that •OH, $O_{2}^{•-}$, and ^1^O_2_ were all generated within the O_3_/SPC system, which was consistent with the ROS identification by quenching experiments.

Based on the results discussed above, the mechanism of SCP degradation by the O_3_/SPC system was speculated as follows: SPC dissolves in water, producing $CO_{3}^{2-}$ and H_2_O_2_, and O_3_ reacts with H_2_O_2_ to generate •OH (Equations (3) and (4)). Afterwards, •OH can be converted to $CO_{3}^{•-}$ and $O_{2}^{•-}$ by further reacting with the $CO_{3}^{2-}$ and H_2_O_2_ that are generated sufficiently (Equations (5)–(9)). Meanwhile, singlet oxygen (^1^O_2_) could be generated, in this case, most likely from the reaction between $O_{2}^{•-}$ and •OH (Equation (20)) [49]. Moreover, the decomposition of O_3_ was considered insignificant due to the neutral pH. Ultimately, the constant formation of ROS in the system can effectively mineralize the SCP fraction into CO_2_ and H_2_O.

(20) $$O_{2}^{•-}+•OH\to \;1O_{2}+{OH}^{-}$$

### 3.4. Identification of SCP Degradation Products with Proposed Degradation Pathways

#### 3.4.1. SCP Degradation Products

The non-selective attack by reactive species in the O_3_/SPC system led to the formation of various degradation intermediates. To determine the structural characteristics of the SCP molecule, DFT analysis was conducted, and the geometry of SCP was optimized at the 6-31G level using Gaussian 09W with the B3LYP method, and its frontier orbital distributions are shown in Figure S8.

To theoretically analyze the location where SCP molecules tend to undergo reactions, the front orbitals of SCP molecules were analyzed using Multiwfn wave function analysis software 3.7, and the contribution value of each atom was calculated, of which the results are demonstrated in Table S1. In the highest occupied molecular orbital (HOMO), the contributions of the S atom (#2, 2.318%), N atoms (#5, 3.063%; #6, 24.278%), and C atoms (#9, 16.255%; #12, 11.504%; #13, 10.426%; #14, 11.041%) were significantly higher than those of other atoms. Compounds containing these sites, characterized by high electron cloud density, are prone to undergoing hydroxylation and methylation through substitution reactions [50]. In the lowest unoccupied molecular orbital (LUMO), the Cl atom (#1, 1.125%), N atoms (#7, 18.233%; #8, 19.618%), and C atoms (#16, 20.877%; #17, 20.091%) exhibited higher contributions, making them preferential sites for nucleophilic attack.

#### 3.4.2. Degradation Pathway Analysis

Figures S9 and S10 display the mass spectra of SCP and its main products during the degradation by O_3_/SPC. Based on the fragment ions marked in the mass spectrogram, the intermediate product may be determined by extracting and evaluating the SCP mass spectrogram. As displayed in Table 1, the transformation products from the degradation of SCP in the O_3_/SPC system are illustrated. The table provides information on the mass-to-charge ratio of the molecular ion detected, the molecular formula, and the proposed structures. The ten most prevalent SCP transformation products were further categorized into three groups based on the abundance of peaks: high, medium, and low. Consequently, P1, P2, P4, P5, and P7 were regarded as the primary SCP oxidation by-products. P1 with *m*/*z* = 130.0161 (C_4_H_4_N_2_Cl) and P2 with *m*/*z* = 146.0121 (C_4_H_4_N_3_OCl) were identified as the major by-products. The presence of other compounds was detected, but their abundance was found to be lower and thus they were considered minor by-products of SPC degradation in the studied processes, which included S3, S6, and S8. It has been established through further research that analogous by-products of SCP decomposition can be produced by multiple oxidation processes, including ozonation, chlorination, permanganate oxidation, and various AOPs, and all the products are expected to be mineralized, forming CO_2_ and H_2_O eventually [51,52], which are anticipated to be of little harm.

**Figure 8:**
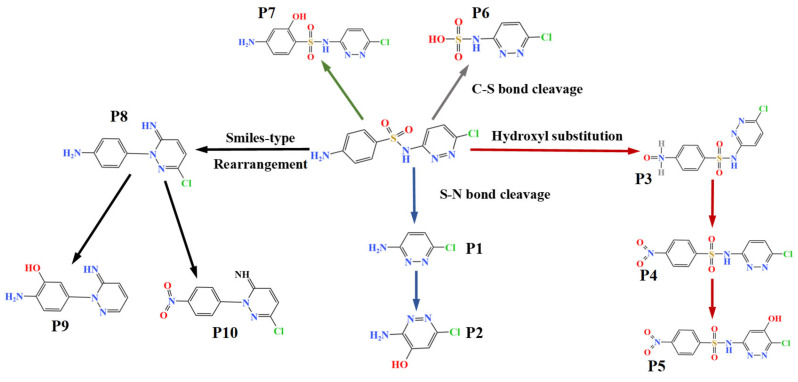
Proposed pathway for SCP degradation by O_3_/SPC system.

Integrating the DFT calculations with the identified intermediates, a comprehensive degradation pathway for SCP in the O_3_/SPC system is proposed (Figure 8). In pathway I, SCP went through a smile-type rearrangement reaction to extrude •SO_2_, forming product P8 (*m*/*z* = 221.0593). Product P8 might have undergone a dehalogenation reaction to produce product P9 (*m*/*z* = 203.1065), which was also reported by He et al. [51] from their experiments on the degradation of SCP in the PS/WS_2_/Fe (III) system. Furthermore, it has been demonstrated that product P8 may undergo oxidation to yield product P10 (*m*/*z* = 251.0308). In pathway II, the attack of •OH and $O_{2}^{•-}$ led to the formation of product P1 (*m*/*z* = 130.0161) by breaking the S-N bond. Subsequent hydroxylation then produced product P2 (*m*/*z* = 146.0121). It was proposed previously that the S-N bond has the smallest population and a relatively long length, which makes it easily destroyed by free radicals [51].

In pathway III, the H atom on the N-H bond undergoes a substitution reaction with •OH, thereby generating the hydroxylamine product P3 (*m*/*z* = 301.0152). This is attributable to the high electronegativity of the N atom, which is a consequence of the substantial π bond in the SCP benzene ring. Then, the N atom on the hydroxylamino group of P3 undergoes electron transfer to form a nitrogen-centered radical, which can be further nitrogenated to form product P4 (*m*/*z* = 314.9931), and the H atom in P4 can be further substituted by a hydroxyl group to form P5 (*m*/*z* = 330.9912). In another way, the H atom on the benzene ring may be attacked by •OH to produce P7 (*m*/*z* = 301.0152) through hydroxylation. Meanwhile, P6 (*m*/*z* = 209.9739) may be generated through the C-S bond, due to its high contribution in HOMO and the longest bond length [53].

### 3.5. Toxicity Evaluation

The formation of various intermediates during oxidation necessitates an evaluation of their potential environmental risks. For this purpose, the ECOSAR software was used in this study to assess and predict the acute and chronic toxicity levels of intermediate degradation products resulting from the degradation of SCP by O_3_/SPC. The Globally Harmonized System (GHS) for Classification and Labeling categorizes toxicity effects into four classes: extremely toxic, toxic, hazardous, and non-hazardous, with the basis for classification shown in Table S2.

As outlined in Table S3, the anticipated acute and chronic toxicity values of SCP and its degradation intermediates are presented. Daphnia magna exhibited the highest sensitivity to SCP, with acute and chronic toxicity levels classified as “toxic” and “extremely toxic”, respectively. In pathway I, the acute toxicity of P1 and P2 was increased in comparison to SCP, as the LC50 of intermediates P1 and P2 was reduced to 0.957 and 1.086, respectively. The toxicity results for P3 and P4 regarding fish and Daphnia magna are classified as “harmless”, whereas the toxicity data for P5 are categorized as “harmful” and “toxic”. Hence, it was indicated that the O_3_/SPC system might have mitigated both acute toxicity and chronic toxicity during SCP degradation. However, considering the current toxicity analysis only targeted the degradation products of the specific pollutant SCP, further variation is needed to assess the variation in comprehensive toxicity in realistic water matrices.

## 4. Conclusions

In this investigation, SPC, activated by O_3_, was proposed as an innovative alternative to H_2_O_2_ for an advanced oxidation process in drinking water, based on the removal performance of the targeted pollutant SCP, which was tested to be affected by SPC dosage, O_3_ dosage, pH, and water substrates. It was observed that the best SCP removal performances were each achieved at a pH of 8, an SPC dose of 20 mg/L, and an O_3_ dose of 4 mg/L, respectively. Furthermore, the water matrix components, including NOM, Cl^−^, and $NO_{2}^{-}$, in water inhibited the oxidation process by competing for reactive species with the pollutant SCP. The ROS, including $O_{2}^{•-}$, ^1^O_2_, and •OH, were identified in SCP degradation based on radical scavenger experiments and EPR tests. The DFT analysis predicted five pathways and ten degradation products for SCP degradation. Toxicity assessment by the ECOSAR software demonstrated that most of the proposed degradation products of SCP exhibited lower levels of toxicity, indicating that the toxicity of SCP may be mitigated by treating with the O_3_/SPC system.

## Figures and Tables

**Table 1:** Retention time (RT), molecular formula, charge-to-mass ratio, and structural formula information for each intermediate product.

Serial Number	Molecular Formula	RT (min)	Structural Formula	*m*/*z*
SCP	C_10_H_9_ClN_4_O_2_S	6.17	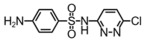	285.0211
P1	C_4_H_4_N_2_Cl	1.5	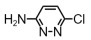	130.0161
P2	C_4_H_4_N_3_OCl	1.73	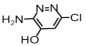	146.0121
P3	C_10_H_9_ClN_4_O_3_S	2.36	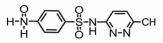	301.0152
P4	C_10_H_7_ClN_4_O_4_S	8.21	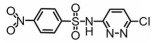	314.9931
P5	C_10_H_7_ClN_4_O_5_S	6.58	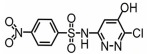	330.9912
P6	C_4_H_4_N_3_SO_3_Cl	1.71	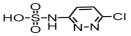	209.9739
P7	C_10_H_9_ClN_4_O_3_S	2.36	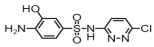	301.0152
P8	C_10_H_9_N_4_Cl	2.66	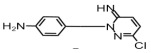	221.0593
P9	C_10_H_10_N_4_O	13.20	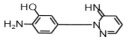	203.1065
P10	C_10_H_9_N_4_Cl	5.36	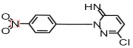	251.0308

## Data Availability

The original contributions presented in this study are included in the article/Supplementary Materials. Further inquiries can be directed to the corresponding authors.

## Supplementary Materials

The following supporting information can be downloaded at: https://www.mdpi.com/article/10.3390/toxics14010073/s1, Figure S1: Experimental device for SCP removal; Figure S2: The fitting curves of degradation rate constants under different O_3_ dosage; Figure S3: The fitting curves of degradation rate constants under different SPC dosage; Figure S4: The fitting curves of degradation rate constants under different pH value; Figure S5: The fitting curves of degradation rate constants under different Cl^−^ concentration; Figure S6: The fitting curves of degradation rate constants under different $NO_{2}^{-}$ concentration; Figure S7: The fitting curves of degradation rate constants under different humic acid concentration; Figure S8: SCP chemical structure: (a) HOMO; (b) LUMO; Figure S9: SCP degradation chromatography of O_3_/SPC; Figure S10: Secondary mass spectrum of SCP degradation intermediates; Table S1: Contribution of SCP molecules to the front orbital; Table S2: Toxicity classification and scope (based on GHS); Table S3: Predicted acute chronic toxicity data for SCP and its degradation products.
