# Supplementary material for: Enhanced Removal of Antibiotic Sulfachloropyridazine in Water Using Sodium Percarbonate Activated by Ozone: Mechanism, Degradation Pathway, and Toxicity Assessment
Source: Toxics. 2026 Jan 13;14(1):73. doi: 10.3390/toxics14010073 (PMC12845579; doi:10.3390/toxics14010073)
Supplement: Supplementary file 1 [file toxics-14-00073-s001.zip › toxics-4060140-supplementary.pdf]

Supplementary material

Enhanced removal of antibiotic sulfachloropyridazine in water using sodium percarbonate activated by ozone: Mechanism, degradation pathway and toxicity assessment

Junqi Jia[a], Wenhao Wang[d], Yulong Liang[e,f], Zhangbin Pan[b*], Congcong Li[b,c*]

a. CAUPD(Beijing) Planning & Design Consultants Co., Ltd., Beijing 100044, China; b. Shandong Province City Water Supply and Drainage Water Quality Monitoring Center, 250021, Jinan, China; c. School of Water Conservancy and Environment， University of Jinan， Jinan 250022， China; d. Jinan Municipal Engineering Design & Research Institute Co., Ltd, 266100, Jinan, China; e. College of Municipal and Environmental Engineering, Shandong Jianzhu University, 250101, Jinan, China; f. The People's Government of Dongzhuang Town, Ningyang County

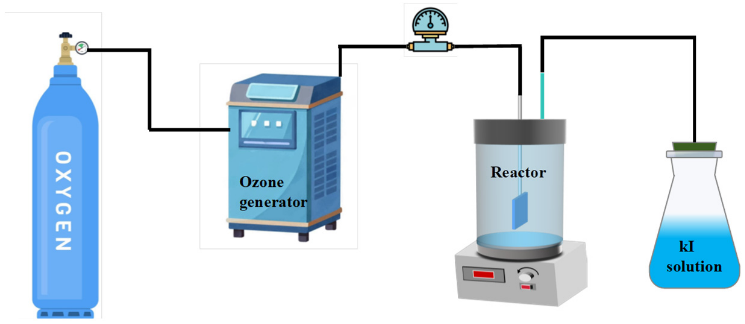

Figure S1 Experimental device for SCP removal

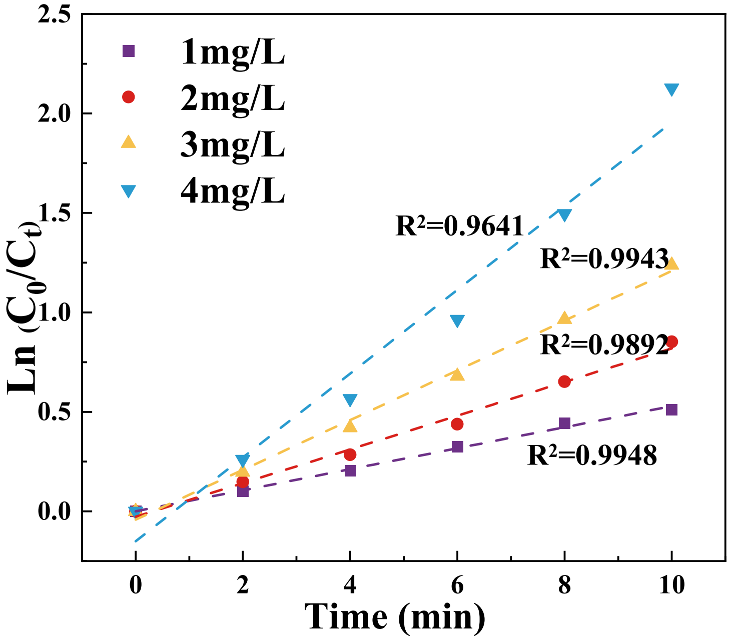

Figure S2 The fitting curves of degradation rate constants under different O_3 dosage

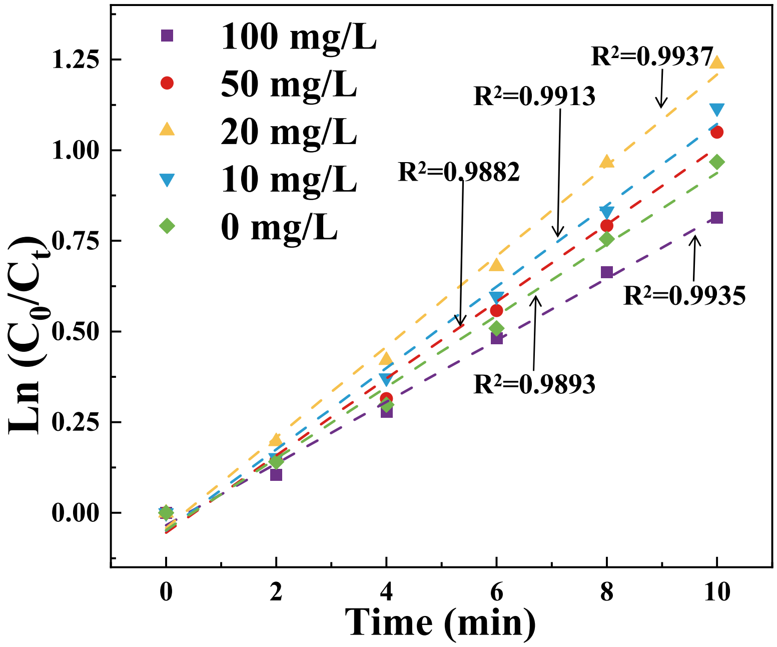

Figure S3 The fitting curves of degradation rate constants under different SPC dosage

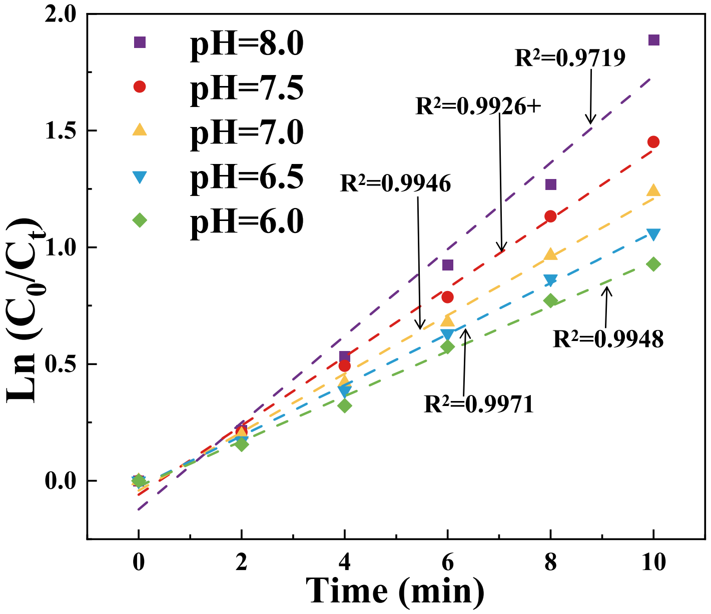

Figure S4 The fitting curves of degradation rate constants under different pH value

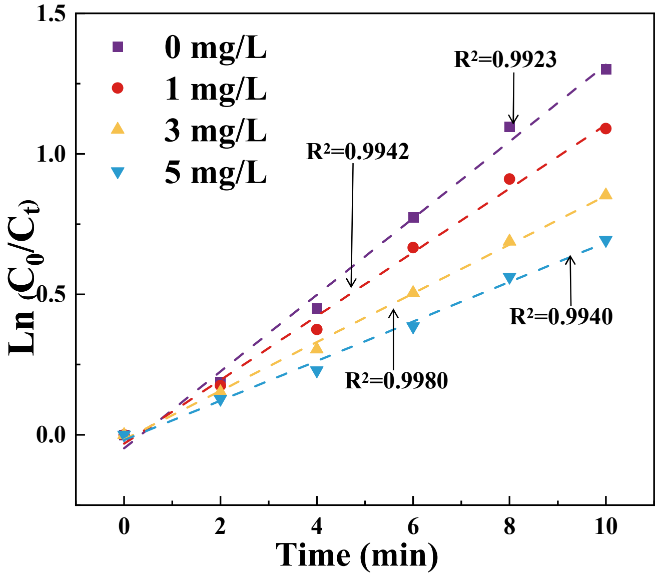

Figure S5 The fitting curves of degradation rate constants under different Cl^- concentration

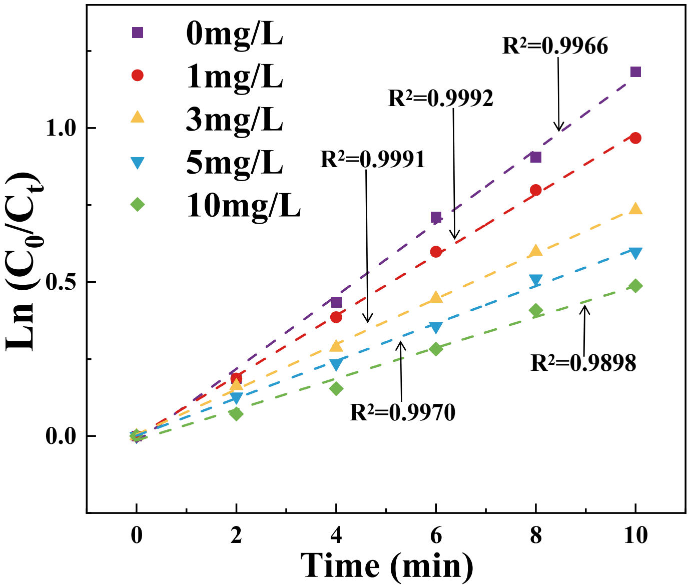

Figure S6 The fitting curves of degradation rate constants under different NO_2^- concentration

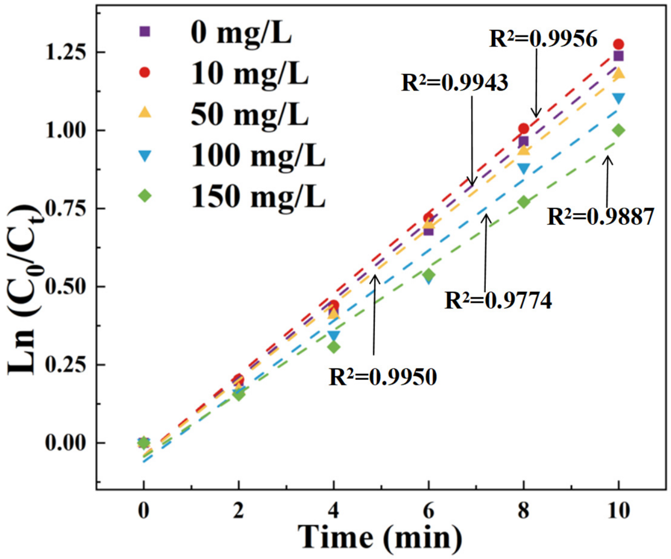

Figure S7 The fitting curves of degradation rate constants under different humic acid concentration

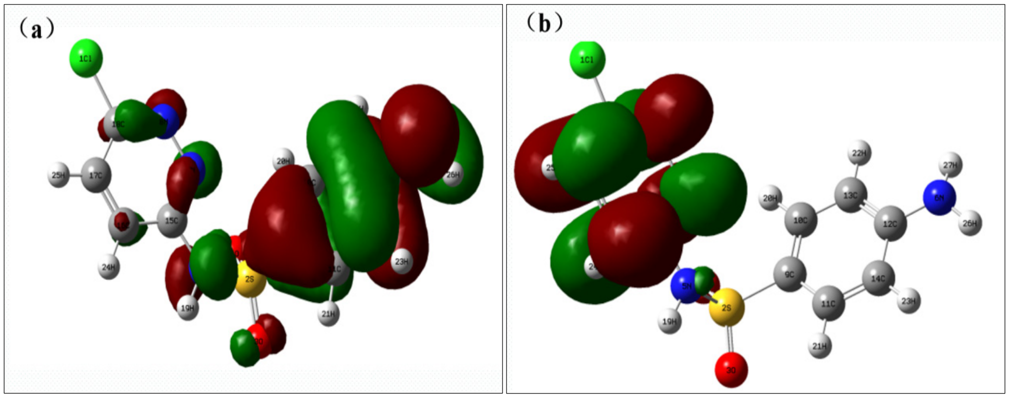

Figure S8 SCP chemical structure: (a) HOMO;(b) LUMO

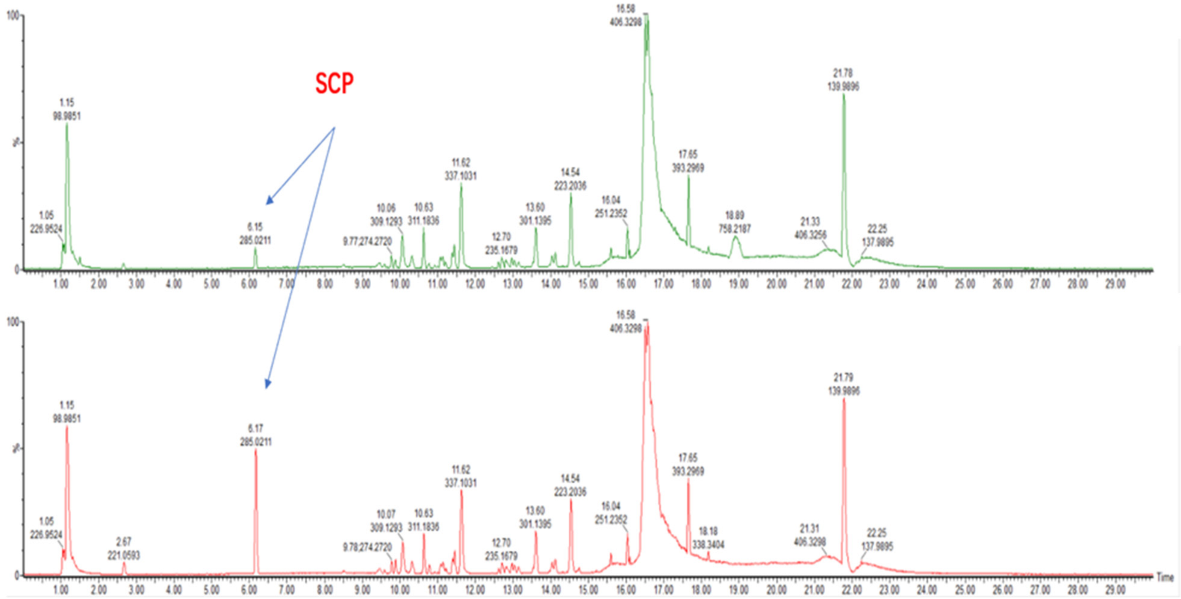

Figure S9 SCP degradation chromatography of O_3/SPC

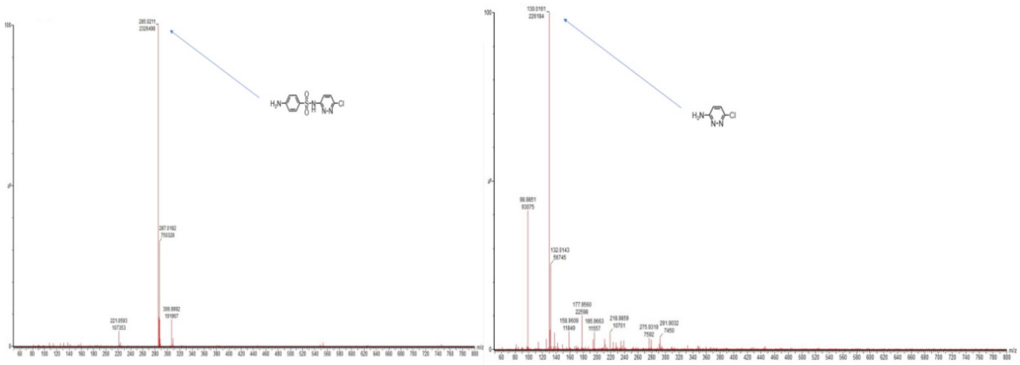

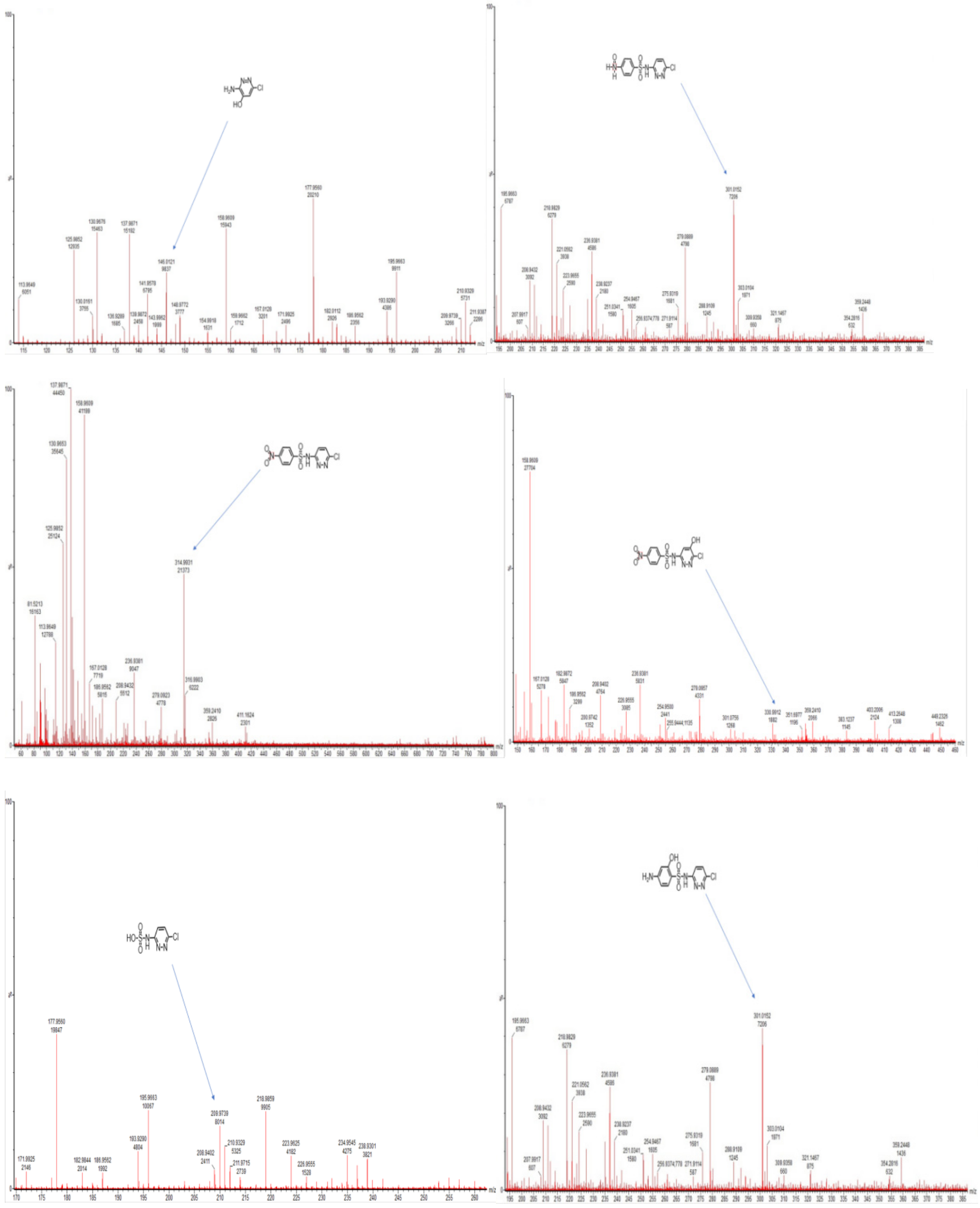

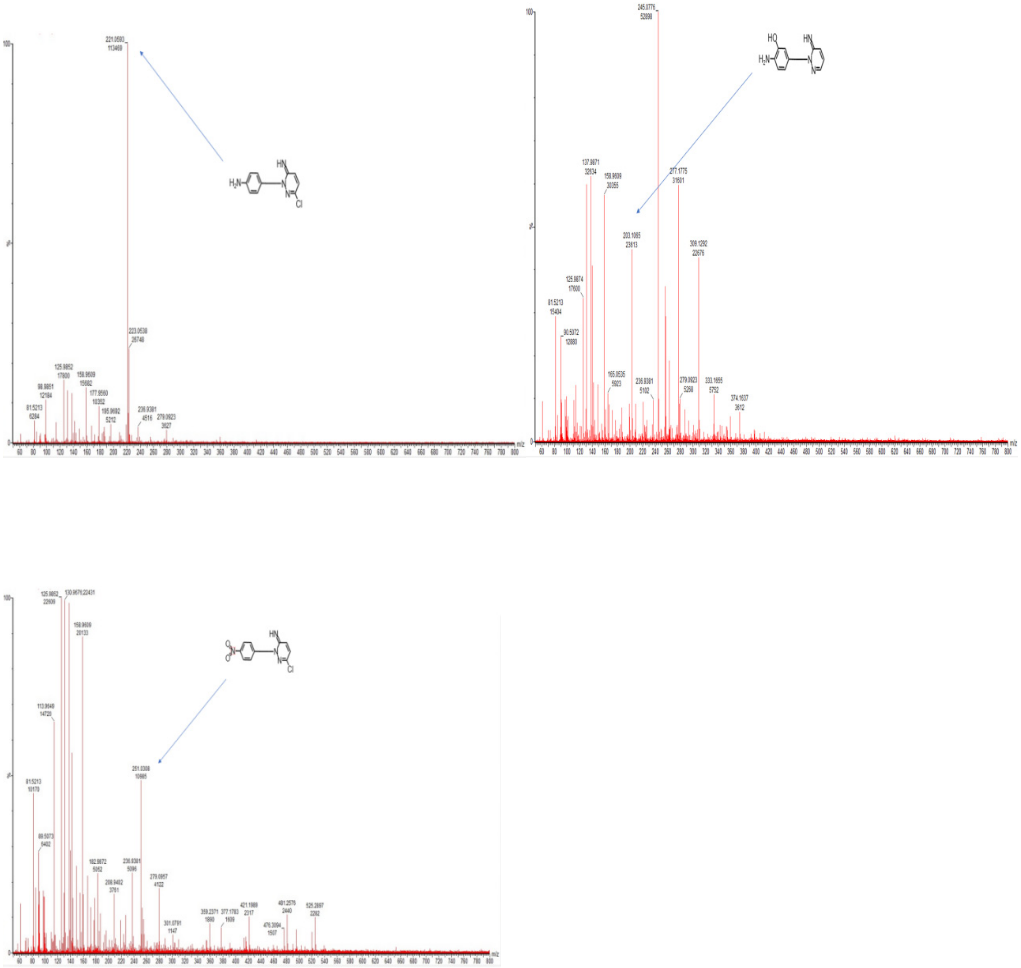

Figure S10 Secondary mass spectrum of SCP degradation intermediates

Table S1 Contribution of SCP molecules to the front orbital

Atom	Contribution rate (%)	
	HOMO orbital	LUMO orbital
1（Cl）	0.386%	1.125%
2（S）	2.318%	0.293%
3（0）	0.898%	0.048%
4（0）	1.561%	0.330%
5（N）	3.063%	0.762%

6（N）	24.278%	0.009%
7（N）	1.629%	18.233%
8（N）	0.873%	19.618%
9（C）	16.255%	0.102%
10（C）	3.614%	0.116%
11（C）	3.764%	0.060%
12（C）	11.504%	0.026%
13（C）	10.426%	0.037%
14（C）	11.041%	0.030%
15（C）	0.551%	5.726%
16（C）	0.471%	20.877%
17（C）	0.129%	20.091%
18（C）	0.621%	6.216%
19（H）	0.298%	0.394%
20（H）	0.171%	0.074%
21（H）	0.186%	0.009%
22（H）	0.760%	0.006%
23（H）	0.822%	0.003%
24（H）	0.049%	3.040%
25（H）	0.026%	2.772%
26（H）	2.153%	0.001%
27（H）	2.154%	0.002%

Table S2 Toxicity classification and scope (based on GHS)

Toxicity range (mg/L)	Toxicity level
$LC_{50}/EC_{50}/ChV \leq 1$	extremely toxic
$1 < LC_{50}/EC_{50}/ChV \leq 10$	toxic
$10 < LC_{50}/EC_{50}/ChV \leq 100$	harmful
$LC_{50}/EC_{50}/ChV > 100$	harmless

Table S3 Predicted acute chronic toxicity data for SCP and its degradation products

Substance Description	Acute toxicity（mg/L）			Chronic toxicity（mg/L）		
	Fish LC50	Daphnia magna LC50	Chlorella EC50	Fish ChV	Daphnia magna ChV	Chlorella ChV
SCP	613.528	2.113	8.194	0.866	0.109	12.666
P1	196.952	0.957	3.309	1.118	0.043	5.225
P2	36.176	1.086	4.837	2.774	0.066	2.101
P3	1451.808	4176.784	35.875	0.998	72.528	15.929
P4	398.574	780.229	11.652	0.442	23.244	7.305
P5	49.887	3.16	22.425	0.769	1.958	3.13
P6	14700000	5020000	460000	67942.766	119000	38950.152
P7	129.403	2.273	13.648	2.193	0.202	6.903
P8	458.86	1.637	6.274	2.629	0.083	9.719
P9	147.684	1.547	12.392	21.783	0.197	7.258
P10	1427.418	743.371	387.317	125.998	56.992	83.667
